# Thermodynamic power of non-Markovianity

**DOI:** 10.1038/srep27989

**Published:** 2016-06-21

**Authors:** Bogna Bylicka, Mikko Tukiainen, Dariusz Chruściński, Jyrki Piilo, Sabrina Maniscalco

**Affiliations:** 1 ICFO-Institut de Ciencies Fotoniques, Mediterranean Technology Park, 08860 Castelldefels (Barcelona), Spain; 2 Institute of Physics, Nicolaus Copernicus University, Grudziądzka 5/7, 87–100 Toruń, Poland; 3 Turku Centre for Quantum Physics, Department of Physics and Astronomy, University of Turku, FI-20014, Turun Yliopisto, Finland

## Abstract

The natural framework to discuss thermodynamics at the quantum level is the theory of open quantum systems. Memory effects arising from strong system-environment correlations may lead to information back-flow, that is non-Markovian behaviour. The relation between non-Markovianity and quantum thermodynamics has been until now largely unexplored. Here we show by means of Landauer’s principle that memory effects control the amount of work extraction by erasure in presence of realistic environments.

Since the dawn of Quantum Mechanics, understanding the elusive border between the quantum world and the emergent classical one has been considered the quantum Holy Grail. While we still do not have a final answer to this question, we have certainly gained insight on the physical phenomena responsible for the quantum-to-classical transition. The theory of environment induced decoherence allows one to understand the loss of quantum properties in terms of information about the open system which is continuously lost into the environment. The tendency of quantum features to decay can therefore be traced back to the fragility of quantum information encoded in open quantum systems^1^.

The specific details of this dynamical process depend on a number of properties of both system and environment. However, for non-Markovian open quantum systems, memory effects allow for a partial return of the information previously lost into the environment and may even lead to information trapping^2^,^3^,^4^,^5^,^6^,^7^,^8^,^9^,^10^,^11^,^12^,^13^,^14^. But what do we exactly mean with loss of information or information back-flow? And, what are the thermodynamical implications of the revivals or trapping of quantum information?

In quantum physics, the lack of knowledge on a system is generally quantified by means of von Neumann entropy. The very same quantity appears in the quantum extension of Landauer’s erasure principle connecting thermodynamics and information theory^15^,^16^. One would be therefore tempted to identify information back-flow with partial decrease of von Neumann entropy of the system and use Landauer’s principle to elaborate on the implications in thermodynamics. Unfortunately, there is no connection between the behaviour of von Neumann entropy and the non-Markovian character of the open system dynamics. The von Neumann entropy of an open system may indeed decrease in time even if its quantumness, e.g., the amount of entanglement, decays and irrespectively of the presence of information back flow. Memory effects induced by the environment have a much more subtle origin.

The first attempt to rigorously quantify memory effects, and hence non-Markovianity, in terms of information flow used the concept of distinguishability between states^2^. Since this first seminal manuscript several non-Markovianity indicators have been proposed in the literature, based on different ways to quantify information^5^,^6^,^8^,^9^,^10^,^11^,^12^,^13^,^14^. In general these indicators do not coincide: defining quantum information is indeed a tricky business.

A crucial point is that any good measure of non-Markovianity must satisfy what is know as contractivity property of the dynamics. Physically, this means that the initial value of a certain quantity satisfying such property should always remain greater than its value at a subsequent time of the dynamics, when it is measured. A model-independent link between thermodynamical quantities and memory effects therefore requires that the given thermodynamical quantity is contractive during any physical time evolution. This automatically rules out not only von Neumann entropy, but also heat, energy, work, as standardly defined in thermodynamics, and any related quantities.

In this Article we show that the proper approach to connect non-Markovian memory effects and the evolution of thermodynamical quantities requires the description not only of the system, whose information content we are interested in, but also of an observer. Interestingly, quantum mechanical correlations between system and observer may lead to the exciting possibility of extracting work while erasing information on the system^17^,^18^. We will show that memory effects are instrumental in the persistence of work extraction by erasure in presence of realistic environments, in particular in the case of unital dynamics.

## Non-Markovianity Measures

Recent investigations on open quantum systems highlight the existence of two different classes of dynamical behavior: Markovian and non-Markovian quantum dynamics. Historically, in the quantum domain Markovian dynamics has been associated to the semigroup property of the dynamical map describing the system evolution. If one thinks in terms of a microscopic model of system, environment and interaction, a Markovian description of the open system requires a number of assumptions, such as system-reservoir weak coupling, and leads to the so-called Lindblad master equation^19^,^20^. In certain physical contexts, however, such approximations are not justified and one needs to go beyond perturbation theory. One of the first features that emerges from the analysis of exact models is that memory effects, usually associated to recoherence and information backflow, do not occur only when the semigroup property of the dynamical map is not satisfied, but they rather characterise non-divisible dynamical maps. In this spirit, a non-Markovianity measure quantifying the deviation from divisibility has been proposed in ref. 5. This measure is based on a mathematical property of the dynamical map and its physical interpretation has been only very recently fully unveiled^21^.

A different perspective on the definition of non-Markovianity is to interpret memory effects in terms of information back-flow. This path was firstly undertaken by Breuer, Laine and Piilo by quantifying the information content of an open quantum system in terms of distinguishability between pairs of states^2^. Presenting all the recent information-theoretic measures of non-Markovianity is beyond the purpose of this section. We refer the interested reader to two recent reviews that fully cover this topic^13^,^14^. The main motivations which have led to the new definitions of non-Markovianity are the following: (i) to give a physical interpretation of memory effects in terms of information back-flow; (ii) to define non-Markovian dynamics independently from the specific structure of the master equation of the system. The underlying idea is to have a definition that is not based on mathematical properties but rather on the occurrence of physical effects, such as revivals of the information content of a quantum open system.

The key property exploited in these definitions is that the time evolution of a given quantifier of information suitable to describe memory effects, e.g. distinguishability between quantum states, is contractive under CPTP maps. Hence a temporary increase of distinguishability, which is physically interpreted as a partial increase in the information content of the open system due to memory effects, always implies that divisibility of the dynamical map is violated. In more detail, let us indicate with 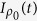 a quantifier of the information content of the system. This generally depends on the initial state *ρ*_0_ (or in some cases on pairs of initial states) and, due to contractivity, it is such that 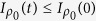, for any *t* ≥ 0. A non-Markovianity measure 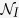 is now defined as
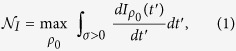
where the integral is defined over all time intervals for which 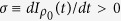. The quantity *σ* defines information flow. Hence, information back-flow is indicated by positive values of the derivative of 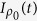. As an example, in case of the measure of Luo *et al*.^8^, which plays a key role in our Article, 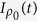 is the mutual information between system and ancilla when these two quantum systems are initially prepared in a maximally entangled state, in the spirit of the Choi-Jamiolkowski isomorphism^22^. Since in the measure of Luo *et al*. the initial state of the system-ancilla composite system is fixed, an optimisation over the initial states of the system is not required. It is worth mentioning that, while the different non-Markovianity measures in general do not coincide, for a qubit undergoing pure dephasing (e.g., the Ising model example considered in the following) they all consistently witness non-Markovian behaviour^14^.

## Work Extraction by Erasure

Work extraction protocols in individual quantum systems have recently received a great deal of attention^18^,^23^,^24^,^25^. When one tries to ascertain properties of a quantum system *S* it is natural to introduce an observer which performs measurements on *S*. In this framework the amount of information stored in a system is observer-dependent. The fully quantum scenario is described by a situation in which the observer possesses a quantum memory *Q*. It has been shown^18^ that in the thermodynamic limit, the work cost of erasure, i.e., the work needed to erase the information stored in *S*, is given by
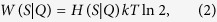
and the extractable work, i.e., the work that one can extract from an *n*-qubit system, is *W*_*ex*_ = [*n* − *H*(*S* | *Q*)]*kT* ln 2. In the equations above *k* is the Boltzmann constant, *T* is the temperature of the reservoir used for the optimal erasure process, and *H*(*S* | *Q*) = *H*(*SQ*) − *H*(*Q*) is the conditional entropy, i.e., the entropy of the system conditioned on the memory, with *H*(*SQ*) and *H*(*Q*) the von Neumann entropies of the combined system-memory and of the memory, respectively. The conditional entropy may be recast in the form *H*(*S* | *Q*) = *H*(*S*) − *I*(*S* : *Q*), where the quantum mutual information *I*(*S* : *Q*) = *H*(*S*) + *H*(*Q*) − *H*(*SQ*) quantifies the amount of total (quantum and classical) correlations shared by *S* and *Q*^26^,^27^. If the system and the memory are prepared in a quantum correlated state, such as a maximally entangled state, the conditional entropy may be negative. When this happens one can extract work while erasing information on the system. This is a genuinely quantum phenomenon with no classical counterpart.

We conclude this section by briefly reviewing the optimal protocol for the work extraction by erasure^18^. In this setting the observer *Q* possessing the quantum memory erases information on the system by using a heat bath at temperature *T*. The observer can store and withdraw energy from a battery and the rest of the universe is represented as a reference system. More precisely, the reference system models all systems other than *S* that can have information on the memory. Therefore, in our description, the reference system will generally contain also the environment affecting the memory.

The optimal erasure protocol consists of the following three steps^18^.

(Step 1) We start by manipulating the system *S* in order to compress the correlations between the system and the memory into a pure state of a subsystem *X* of *S* ⊗ *Q*. We assume that the Hamiltonian of the system-memory -composite *S* ⊗ *Q* is fully degenerate. This state is maximally entangled between two subsystems of *S* ⊗ *Q*. Note that this process can be carried out by using local reversible transformations, i.e., local unitaries, on *S*. We assume that these transformations are implemented in a time scale much shorter than *τ*_*d*_. The compression of correlations of step 1 can be performed for any generic state of system and memory, therefore also for the generally mixed state resulting from a non-unitary evolution of either system or memory due to the real environment, as considered in our Article. We refer the reader to ref. 18 for all details.

(Step 2) We use the pure state of the subsystem *X* to extract roughly [*n* − *H*(*S* | *Q*)]*kT* ln 2 of work, with *n* the size of the system. This process uses a heat bath at temperature *T* and a battery. The extracted work is stored as energy difference Δ*E* in the charge of the battery. This is the most delicate part of the protocol as it requires an isothermal quasistatic process and we need to assume that its duration is overall shorter than *τ*_*d*_. The total duration of this step will be inversely proportional to the temperature *T* of the bath, its size with respect to the *S* ⊗ *Q* composite system, and the coupling strength between the bath and subsystem *X*. As we can arbitrarily choose the heat bath used in the work extraction protocol, we can assume to work in a regime in which the finite duration of this step is smaller than *τ*_*d*_ and the errors due to non-adiabaticity of the process are negligible. At the end of the process the initially maximally entangled state of the subsystem *X* is left in a maximally mixed state.

(Step 3) Finally, we erase system *S* by bringing it to a state |0〉. This step follows the inverse procedure of step 2 and requires an energy investment of *nkT* ln 2: the reduction comes from the energy of the battery. The assumptions regarding the relevant time scales are the same as in step 2. Since the state at the beginning of step 2 is maximally entangled, the battery will be left with *H*(*S* | *Q*)*kT* ln 2 energy, which corresponds to work extracted during the whole erasure protocol.

## Results

**Figure 1:**
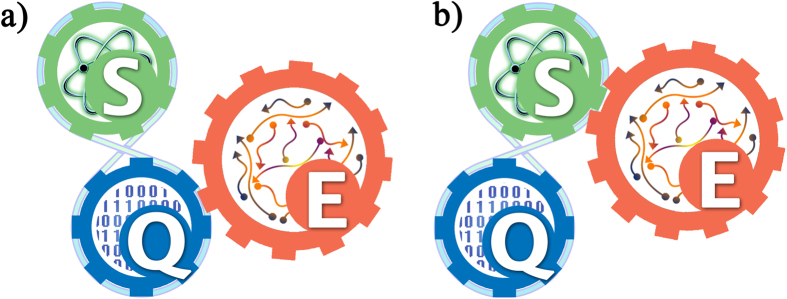
Illustration of the two physical scenarios considered in the Article. We consider a system *S* given access to a quantum memory *Q* under natural conditions: in (**a**), the memory *Q* is subject to interaction with an environment *E* (open quantum memory); in (**b**) it is the system *S* that is coupled with the environment (open quantum system).

As always, when dealing with a quantum-enhanced phenomenon, it is worth investigating how resistant is the entanglement-powered work extraction to the unavoidable environmental noise. Our aim is to tackle this question in full generality by looking at the two most fundamental scenarios. We consider the case in which either the system or the memory is subjected to the action of the environment. These two situations are pictorially illustrated in Fig. 1. The action of the environment on the system or the memory is described in terms of a dynamical map Φ_*t*_, that is a *t*-parametrized family of completely positive and trace preserving (CPTP) maps.

Both the work cost of erasure and the extractable work are now time-dependent. The interpretation of these quantities in our out-of-equilibrium scenario is based on the ability to extract work optimally, at time *t*, that is after decoherence has taken place. A crucial assumption for the proof of the existence of an optimal erasure protocol is that the joint state of memory and reference is preserved during the erasure process and that the reference system is not touched^18^. This generally implies that during the erasure protocol the evolution of the memory and of the environment should be neglected. We will therefore assume that the typical decoherence timescale *τ*_*d*_, due to the presence of the real environment, is much longer than the total duration of the erasure protocol. Within this assumption the extractable work *W*_*ex*_ is defined as the increase Δ*E* in the average energy of a battery, the conditional entropy *H*(*S* | *Q*) is calculated from the decohered system-memory state at time *t*, and *T* is the temperature of the reservoir used in the optimal erasure protocol. It is worth noticing that, while the assumptions listed above are crucial in the open memory scenario, they can be released when the environment affects the system only. In the latter case, indeed, one does not need to include the real environment within the reference state since, by definition, the environment only acts on the system without affecting the memory.

*Open quantum memory:* We indicate the conditional entropy in equation (2) with *H*(*S* | *Q*_*t*_), with *Q*_*t*_ = Φ_*t*_(*Q*), to emphasize that only *Q* is evolving due to the coupling to the environment. The extractable work can be written as
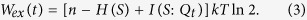


For divisible dynamical maps, namely when Φ_*t*_ = Φ_*t*,*s*_ ⚬ Φ_*s*_, with *s* ≤ *t* and Φ_*t*,*s*_ CPTP, the data processing inequality^27^ implies, that 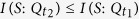, for *t*_1_ ≤ *t*_2_. In this case, the extractable work monotonically decreases in time whereas the work cost of erasure monotonically increases, as one would intuitively expect.

*Open quantum system:* When the environment acts on the system, rather than on the memory, the extractable work is given by
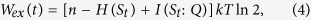
where *H*(*S*_*t*_ | *Q*) indicates the conditional entropy when the system is coupled to the environment. Note that, in this case, the entropy of the system is also evolving in time.

Once again, for divisible dynamics, 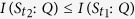, for *t*_1_ ≤ *t*_2_, which implies that an increase in extractable work requires a decrease in entropy of the system. This meets our intuition, since it amounts to saying that the more we know about the system, namely the closer it is to a pure state, the more work we can extract from it. Equivalently, the closer the system is to a maximally mixed state, the smaller is the extractable work. As we will see in the following, however, non-Markovian memory effects may defy this intuition.

We note in passing that for unital dynamical maps, i.e., Φ_*t*_(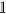) = 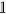, the entropy of the system will not evolve in time when the initial system-memory is prepared in a maximally entangled state. In this case, for both the open quantum system and the open quantum memory scenario, the dynamics of the extractable work, given by equations (3) and (4), only depends on the evolution of the mutual information.

### Thermodynamic meaning of non-Markovianity

In absence of initial correlations between system and environment, the most general form of open system evolution is described by dynamical maps which may violate the divisibility property while still being CPTP. When this happens, the quantum mutual information may temporarily increase for certain time intervals. This, in turn, generally leads to a partial recovery of extractable work or, equivalently, to a decrease in the work cost of erasure.

Recent research on open quantum systems has highlighted the fact that memory effects can be associated to different manifestations of non-Markovianity which, in turn, are revealed by different physical quantities. Several non-Markovianity measures capturing this plethora of phenomena have been introduced and used to describe information back-flow and recoherence^2^,^5^,^6^,^7^,^8^,^9^,^10^,^11^,^12^. Equations (3) and (4) show that a connection between thermodynamical quantities and memory effects can be established in terms of the non-Markovianity indicator introduced by Luo *et al*.^8^.

Specifically, one introduces information flow as the time derivative of the mutual information, in our case *dI*(*S* : *Q*)/*dt*. Both in the open quantum memory and in the open quantum system scenario (for unital maps) non-Markovianity and information back-flow occur whenever, for certain time intervals,
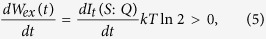
where the time evolution of the mutual information *I*_*t*_(*S* : *Q*) arises from the non-unitary dynamics of either the memory, *I*(*S* : *Q*_*t*_), or the system, *I*(*S*_*t*_ : *Q*). In the open quantum system case, when the dynamical map is non-unital, revivals of extractable work are due to the interplay between memory effects and entropy dynamics, as we will discuss further in one of the examples, since *dW*_*ex*_/*dt* ∝ *dI*(*S*_*t*_ : *Q*)/*dt* − *dH*(*S*_*t*_)/*dt*.

We will now consider three exemplary cases to illustrate our results, namely a qubit coupled to a classical environment (Pauli channel), a spin environment (Ising model) and a bosonic environment (amplitude damping channel). We are interested in the situation in which the qubit system and the memory are initially maximally entangled since this corresponds to the maximal work extraction by erasure scenario.

**Figure 2:**
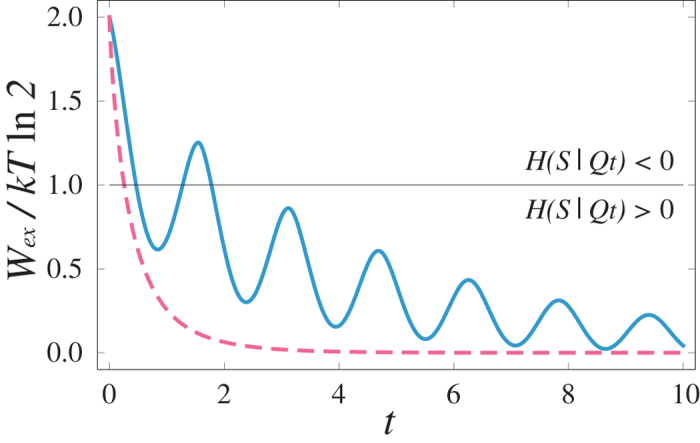
Time evolution of the extractable work *W*_*ex*_/*kT* ln2 for one qubit system. The blue solid line corresponds to a Pauli channel model with decay rates *γ*_1_(*t*) = *γ*_2_(*t*) = *λ*/2, and *γ*_3_(*t*) = *ω* tan(*ωt*)/2, with *λ* = 0.1 and *ω* = 2 (a.u.). The red dashed line corresponds to a Pauli channel model^37^ with decay rates *γ*_1_(*t*) = *γ*_2_(*t*) = *λ*/2, and *γ*_3_(*t*) = −*ω* tanh(*ωt*)/2, with *λ* = 1 and *ω* = 0.5 (a.u.). In both cases the dynamical map is non-divisible but only in one scenario (blue solid line) revivals of work extraction are present.

As a first example we study the time evolution of the extractable work for time-dependent Pauli channels, which are unital. Pauli channels describe depolarising noise acting independently along the *x*, *y*, and *z* directions with time-dependent rates *γ*_1_(*t*), *γ*_2_(*t*) and *γ*_3_(*t*), respectively^11^ (see also the Supplementary Information). We compare two different open dynamics: the corresponding evolutions of the extractable work are given by the solid blue line and the dashed red line in Fig. 2. In both cases the dynamical map is non-divisible, but only in one case oscillations of extractable work do take place. Notably, one can distinguish between quantum and classical memory effects. The former ones are associated to revivals of a uniquely quantum property, i.e., negative conditional entropy, and hence to the reappearance of extractable work by erasure. The latter ones are responsible for revivals of work extraction in the region where the conditional entropy is however positive (See Fig. 2).

### Non-Markovian enhancement of work extraction

As the second example, we consider a physical system consisting of a central spin coupled to a spin chain. More precisely we focus on the Ising model in a transverse field^28^; for details we refer to the Supplementary Information. This systems exhibits a quantum phase transition between ferromagnetic state and paramagnetic state when the parameter λ^*^, measuring the ratio between the strength of the transverse field and the coupling between the environmental spins, equals unity. It has been shown^29^ that the central spin dynamics is non-Markovian for any value of λ^*^, except at phase transition. This is therefore an ideal system to study the modification of the dynamics induced by non-Markovianity as we can control the non-Markovian character via the parameter λ^*^.

**Figure 3:**
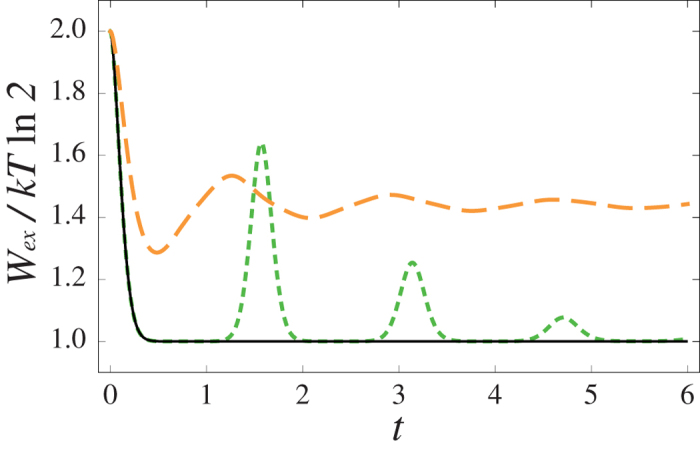
Time evolution of the extractable work of one qubit for an Ising model in a transverse field. We compared the dynamics for *N* = 4000 spins and three values of renormalized transverse field *λ* : λ^*^ = 1 (black solid line), λ^*^ = 0.1 (green short-dashed line) and λ^*^ = 1.9 (orange long-dashed line). The plots show how non-Markovian memory effects allow to retain or regain extractable work, leading to a thermodynamic enhancement compared to the Markovian case (black solid line).

Once again, due to unitality, the extractable work by erasure is just proportional to the mutual information both in the open system and the open memory scenarios. Figure 3 shows the behaviour of extractable work for three exemplary values of λ^*^. For λ^*^ = 1 (black solid line) the extractable work quickly decreases to unity signalling that the initially positive work cost of erasure goes to zero after a short transient time. In this case the spin environment is at quantum phase transition and the dynamics of the central spin is Markovian.

For λ^*^ = 0.1 (green short-dashed line), after decreasing to unity, the extractable work presents oscillations with maximum amplitude damped in time. These oscillations, corresponding to revivals of mutual information, characterize the dynamics when the environment is in its ferromagnetic ground state. Finally, for λ^*^ = 1.9 (orange long-dashed line) oscillations in the extractable work are also present while this quantity remains larger than unity at all the times, meaning that work extraction by erasure is always possible. The example illustrates how non-Markovian memory effects allow to retain or regain extractable work, leading to a thermodynamic enhancement compared to the Markovian case. Interestingly, contrarily to the Pauli channel example previously discussed, in the Ising dynamics the extractable work always remains positive because classical correlations between the system and the memory are never destroyed.

### The power of quantum correlations

**Figure 4:**
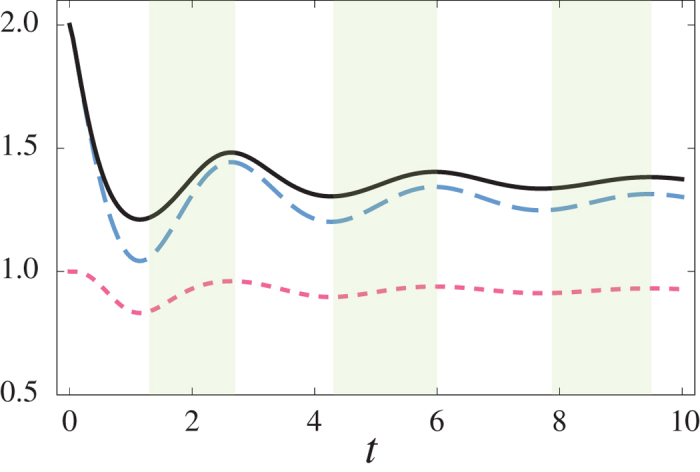
Time evolution of the extractable work (solid black line), the mutual information (blue long-dashed line) and system entropy (red short-dashed line), for the amplitude damping photonics band gap model, with detuning from the band gap edge frequency *δ* = −1 (a.u.). The green shaded regions highlight time intervals in which one observes a partial recovery of the extractable work even though the system entropy increases.

The third example highlights the interesting situation in which memory effects are responsible for a counter-intuitive behaviour of the extractable work. In the open quantum system scenario the time evolution of the extractable work is given by equation (4). When the open system dynamics is Markovian, the mutual information monotonically decreases. Hence, in this case an increase of the extractable work can only occur when the entropy of the system decreases. Non-Markovian dynamics, however, allows for revivals of the extractable work even when the entropy of the system is constant or increasing. Figure 4 illustrates the dynamics of extractable work, as defined in equation (4), mutual information and system entropy for an amplitude damping photonic band gap model^12^ (See the Supplementary Information). In the figure, the green shaded regions highlight time intervals in which one observes a partial recovery of the extractable work even though the system entropy increases. This apparent contradiction is in fact resolved by noticing that, in this setting, what counts is the amount of information on the system possessed by an observer with a quantum memory. Therefore, it is clear that an increase in the system-memory correlations, indicated by the behaviour of the mutual information, plays a key role in determining the extractable work.

## Discussion

The interpretation of non-Markovian memory effects in terms of revivals of either the work cost of erasure or the extractable work unveils a fundamental link between the theory of open quantum systems and quantum thermodynamics. Such a connection also gives a powerful answer to the question of the potential usefulness of non-Markovianity^30^,^31^,^32^, when we think in terms of reservoir engineering^33^,^34^,^35^. Recent advances in reservoir engineering techniques have indeed proven that, although we cannot ultimately get rid of the real environment causing the disappearance of quantum properties, we do have a certain degree of controllability on some of its properties such has its spectral density. We can therefore think of optimal reservoirs for preservation of quantum properties such as negative conditional entropy. This in turn would have immediate consequences for technologies operating at the Landauer limit.

Perhaps the widest and most far-reaching impact would be connected to computer technology. One of the main obstacle to miniaturisation of computing devices is indeed the heat generated by computation. As all irreversible computations can be decomposed into reversible computations followed by erasure, the problem of heating can be traced back to the cost of erasure^36^. When computation is carried on by quantum systems, it is even possible to erase information by cooling the environment. This possibility does not last forever, but only for a short transient whose length, however, can be manipulated by reservoir engineering. Our results indeed show that memory effects lead to revivals of extractable work and work cost of erasure, hence controlling the heat generated during the computation.

## Additional Information

**How to cite this article**: Bylicka, B. *et al*. Thermodynamic power of non-Markovianity. *Sci. Rep.* **6**, 27989; doi: 10.1038/srep27989 (2016).

## Supplementary Material

Supplementary Information

## References

[b1] Zurek W. H. Decoherence, einselection, and the quantum origins of the classical. Rev. Mod. Phys. 75, 715–765 (2003).

[b2] Breuer H.-P., Laine E.-M. & Piilo J. Measure for the degree of non-Markovian behavior of quantum processes in open systems. Phys. Rev. Lett. 103, 210401 (2009). 20366019 10.1103/PhysRevLett.103.210401

[b3] Piilo J., Maniscalco S., Härkönen K. & Suominen K.-A. Non-Markovian quantum jumps. Phys. Rev. Lett. 100, 180402 (2008). 18518352 10.1103/PhysRevLett.100.180402

[b4] Wolf M. M., Eisert J., Cubitt T. S. & Cirac J. I. Assessing non-Markovian quantum dynamics. Phys. Rev. Lett. 101, 150402 (2008). 18999575 10.1103/PhysRevLett.101.150402

[b5] Rivas A., Huelga S. F. & Plenio M. B. Entanglement and Non-Markovianity of Quantum Evolutions. Phys. Rev. Lett. 105, 050403 (2010). 20867898 10.1103/PhysRevLett.105.050403

[b6] Vasile R., Maniscalco S., Paris M. G. A., Breuer H.-P. & Piilo J. Quantifying non-Markovianity of continuous-variable Gaussian dynamical maps. Phys. Rev. A 84, 052118 (2011).

[b7] Lu X.-M., Wang X. & Sun C. P. Quantum Fisher information flow and non-Markovian processes of open systems. Phys. Rev. A 82, 042103 (2010).

[b8] Luo S., Fu S. & Song H. Quantifying non-Markovianity via correlations. Phys. Rev. A 86, 044101 (2012).

[b9] Liu B.-H. . Experimental control of the transition from Markovian to non-Markovian dynamics of open quantum systems. Nature Phys . 7, 931–934 (2011).

[b10] Lorenzo S., Plastina F. & Paternostro M. Geometrical characterization of non-Markovianity. Phys. Rev. A 88, 020102(R) (2013).

[b11] Chruściński D. & Maniscalco S. Degree of Non-Markovianity of Quantum Evolution. Phys. Rev. Lett. 112, 120404 (2014). 24724632 10.1103/PhysRevLett.112.120404

[b12] Bylicka B., Chruściński D. & Maniscalco S. Non-Markovianity and reservoir memory of quantum channels: a quantum information theory perspective. Scientific Reports 4, 5720 (2014). 25043763 10.1038/srep05720 PMC4104480

[b13] Rivas A., Huelga S. F. & Plenio M. B. Quantum non-Markovianity: characterization, quantification and detection. Rep. Prog. Phys. 77, 094001 (2014). 25147025 10.1088/0034-4885/77/9/094001

[b14] Breuer H.-P., Laine E.-M., Piilo J. & Vacchini B. Non-Markovian dynamics in open quantum systems. Rev. Mod. Phys. 88, 021002 (2016).

[b15] Landauer R. Irreversibility and heat generation in the computing process. IBM J. Res. Develop. 5, 183–191 (1961).

[b16] Berut A. . Experimental verification of Landauer’s principle linking information and thermodynamics. Nature 483, 187–189 (2012). 22398556 10.1038/nature10872

[b17] Maruyama K., Nori F. & Vedral V. The physics of Maxwell’s demon and information. Rev. Mod. Phys. 81, 1–23 (2009).

[b18] del Rio L., Åberg J., Renner R., Dahlsten O. & Vedral V. The thermodynamic meaning of negative entropy. Nature 474, 61–63 (2011). 21637254 10.1038/nature10123

[b19] Lindblad G. On the generators of quantum dynamical semigroups. Commun. Math. Phys. 48, 119–130 (1976).

[b20] Gorini V., Kossakowski A. & Sudarshan E. C. Completely positive dynamical semigroups of N-level systems. J. Math. Phys . 17, 821 (1976).

[b21] Bylicka B., Johansson M. & Acin A. Equivalence between completely-positive-divisibility and information flow. arXiv:1603.04288.

[b22] Choi M.-D. Completely positive linear maps on complex matrices. Lin. Alg. Appl . 10, 285–290 (1975).

[b23] Aberg J. Truly work-like work extraction via a single-shot analysis. Nat. Commun. 4, 1925 (2013). 23801350 10.1038/ncomms2712

[b24] Horodecki M. & Oppenheim J. Fundamental limitations for quantum and nanoscale thermodynamics. Nat. Commun. 4, 2059 (2013). 23800725 10.1038/ncomms3059

[b25] Skrzypczyk P., Short A. J. & Pospescu S. Work extraction and thermodynamics for individual quantum systems. Nat. Commun. 5, 4185 (2014). 24969511 10.1038/ncomms5185

[b26] Hayden P. Quantum information: Entanglement as elbow grease. Nature 474, 41–42 (2011). 21637248 10.1038/474041a

[b27] Wilde M. M. Quantum Information Theory (Cambridge Univ. Press, New York, 2013).

[b28] Quan H. T., Song Z., Liu X. F., Zanardi P. & Sun C. P. Decay of Loschmidt Echo Enhanced by Quantum Criticality. Phys. Rev. Lett. 96, 140604 (2006). 16712060 10.1103/PhysRevLett.96.140604

[b29] Haikka P., Goold J., McEndoo S., Plastina F. & Maniscalco S. Non-Markovianity, Loschmidth echo, and criticality: A unified picture. Phys. Rev. A 85, 060101(R) (2012).

[b30] Vasile R., Olivares S., Paris M. G. A. & Maniscalco S. Continuous-variable quantum key distribution in non-Markovian channels. Phys. Rev. A 83, 042321 (2011).

[b31] Chin A. W., Huelga S. F. & Plenio M. B. Quantum metrology in non-Markovian environments. Phys. Rev. Lett. 109, 233601 (2012). 23368199 10.1103/PhysRevLett.109.233601

[b32] Laine E.-M., Breuer H.-P. & Piilo J. Nonlocal memory effects allow perfect teleportation with mixed states. Scientific Reports 4, 4620 (2014). 24714695 10.1038/srep04620 PMC3980228

[b33] Barreiro J. T. . Experimental multiparticle entanglement dynamics induced by decoherence. Nature Phys . 6, 943–946 (2002).

[b34] Verstraete F., Wolf M. M. & Cirac J. I. Quantum computation and quantum-state engineering driven by dissipation. Nature Phys . 5, 633–636 (2009).

[b35] Biercuk M. J. . Optimized dynamical decoupling in a model quantum memory. Nature 458, 996–1000 (2009). 19396139 10.1038/nature07951

[b36] Bennett C. H. Logical reversibility of computation. IBM J. Res. Dev . 17, 525–532 (1973).

[b37] Hall M. J. W., Cresser J. D., Li L. & Andersson E. Canonical form of master equations and characterization of non-Markovianity. Phys. Rev. A 89, 042120 (2014).

