# Supplementary material for: Thermodynamic power of non-Markovianity
Source: Sci Rep. 2016 Jun 21;6:27989. doi: 10.1038/srep27989 (PMC4914965; doi:10.1038/srep27989)
Supplement: Supplementary Information [file srep27989-s1.pdf]

Supplemental Information: Thermodynamic power of non-Markovianity

Bogna Bylicka[1], Mikko Tukiainen[2], Dariusz Chruściński[3], Jyrki Piilo[2], and Sabrina Maniscalco[2]

[1]*ICFO-Institut de Ciencies Fotoniques, Mediterranean Technology Park,*

08860 Castelldefels (Barcelona), Spain

[2]*Turku Centre of Quantum Physics, Department of Physics and Astronomy,*

University of Turku, FIN-20014 Turku, Finland

[3]*Institute of Physics, Nicolaus Copernicus University,*

Grudziądzka 5/7, 87–100 Toruń, Poland

In this Supplementary Information we present in detail the mathematical description of the physical models used as examples in the main Article. In all the examples we choose as initial state of the system S and quantum memory Q the maximally entangled two-qubit state, $|SQ\rangle = \frac{1}{\sqrt{2}}(|10\rangle - |01\rangle)$. The reason for this choice is that maximally entangled states maximise the amount of work that one can extract from the system [1].

In the Article we investigate two open quantum scenarios, in which either the quantum memory qubit or the system qubit is subjected to environmental noise and therefore undergoing a non-unitary evolution. In general these two situations have different implications on the time evolution of the extractable work. However, if the dynamical map is unital, and the reduced state of the system is maximally mixed (which is true for all the studied examples), then the behaviour in these two scenarios is similar: in both cases the extractable work follows the temporal behaviour of the quantum mutual information.

Two of the analysed physical models, Pauli channels and Ising model, are unital. The last example is an amplitude damping channel, which is non-unital. Hence, we can study the more complex situation when also the system entropy is evolving, and witness the surprising behaviour of the extractable work in time.

It is worth stressing that, due to additivity of quantum mutual information, our examples can be straightforwardly extended to the case of an n-qubit system coupled to an n-qubit memory, where each individual qubit S_i is maximally entangled to their respective memory qubit Q_i and either the system or the memory evolves according to the dynamical map Φ_t describing the action of a local environment. Namely, the full dynamics of the composite system is given by either $\bigotimes_{i=1}^n \mathbb{I}^{S_i} \otimes \Phi_t^{Q_i}(S_iQ_i)$ or $\bigotimes_{i=1}^n \Phi_t^{S_i} \otimes \mathbb{I}^{Q_i}(S_iQ_i)$. In this case, for all the three models of environment considered, the extractable work is just n times the expression plotted in Fig. 2, Fig. 3 and Fig. 4 in the main Article.

I. PAULI CHANNEL

As the first example of a non-Markovian qubit channel, we consider a Pauli channel with dynamical map of the form

$$\Phi_t(\rho) = \sum_{\alpha=0}^{3} p_\alpha(t)\, \sigma_\alpha \rho \sigma_\alpha, \tag{S1}$$

where $0 \le p_\alpha \le 1$ satisfy $\sum_\alpha p_\alpha = 1$, and σ_α are the Pauli matrices ($\sigma_0 = \mathbb{1}$). A local generator L_t corresponding to this channel is defined via

$$L_t(\rho) = \sum_{i=1}^{3} \gamma_i(t)\,(\sigma_i \rho \sigma_i - \rho), \tag{S2}$$

where the relation between probability distribution $p_\alpha(t)$ and local decoherence rates $\gamma_k(t)$ is given by [2]

$$\begin{aligned}
p_0(t) &= \frac{1}{4}[1 + \lambda_1(t) + \lambda_2(t) + \lambda_3(t)],\\
p_1(t) &= \frac{1}{4}[1 + \lambda_1(t) - \lambda_2(t) + \lambda_3(t)],\\
p_2(t) &= \frac{1}{4}[1 - \lambda_1(t) + \lambda_2(t) - \lambda_3(t)],\\
p_3(t) &= \frac{1}{4}[1 - \lambda_1(t) - \lambda_2(t) + \lambda_3(t)],
\end{aligned} \tag{S3}$$

and the time-dependent eigenvalues $\lambda_k(t)$ read

$$\begin{aligned}
\lambda_1(t) &= \exp(-2[\Gamma_2(t) + \Gamma_3(t)]),\\
\lambda_2(t) &= \exp(-2[\Gamma_1(t) + \Gamma_3(t)]),\\
\lambda_3(t) &= \exp(-2[\Gamma_1(t) + \Gamma_2(t)]),
\end{aligned} \tag{S4}$$

with $\Gamma_k(t) = \int_0^t \gamma_k(\tau) d\tau$. A Pauli channel is divisible if and only if $\gamma_k(t)$ satisfy the following conditions

$$\gamma_1(t) \ge 0, \quad \gamma_2(t) \ge 0, \quad \gamma_3(t) \ge 0, \tag{S5}$$

for all $t \ge 0$.

One easily verifies that any Pauli channel is unital, that is, $\Phi_t(\mathbb{1}) = \mathbb{1}$. Since the initial reduced states of both S and Q are maximally mixed, this implies that the reduced system entropies do not evolve under these dynamics; in particular $H(S_t) = H(S) = 1$. Therefore, the time evolution of the extractable work for the one qubit system ($n = 1$) is simply given by

$$W_{ex}(t) = I(S:Q)_t\, kT \ln 2, \tag{S6}$$

where the time evolving mutual information $I(S:Q)_t$ equals to either $I(S:Q_t)$ or $I(S_t:Q)$ in the open memory or open system case, respectively; see equations (3) and (4) of the Article. In this Pauli channel example, the mutual information $I(S:Q_t)$ for the initial state $|SQ\rangle = \frac{1}{\sqrt{2}}(|10\rangle - |01\rangle)$ is easily analytically solved:

$$I(S:Q_t) = 2 + \sum_\alpha p_\alpha(t) \log_2(p_\alpha(t)). \tag{S7}$$

In the Article we analyse, and depict in Fig. 2, the time evolution of the extractable work corresponding to two cases of Pauli channels with different values of the γ-rates. For the first example we choose $\gamma_1(t) = \frac{\lambda}{2} = \gamma_2(t)$ and $\gamma_3(t) = \frac{\omega}{2}\tan(\omega t)$ that clearly violate the divisibility condition of equation (S5) and show revivals in the extractable work (see the solid blue line in Fig. 2 of the main Article). For comparison, as the second example we choose the case analysed in Refs. [2, 3], $\gamma_1(t) = \frac{\lambda}{2} = \gamma_2(t)$ and $\gamma_3(t) = -\frac{\omega}{2}\tanh(\omega t)$. A Pauli channel with such γ-rates also leads to legitimate non-divisible evolution whenever $0 \leq \omega \leq \lambda$, however in this case no revivals of the extractable work are present (see the red dashed line in Fig. 2). This is to show that sheer non-divisibility of the evolving channel does not always imply increasing work gain.

Interestingly, the second example corresponding to $\gamma_3(t) = -\frac{\omega}{2}\tanh(\omega t)$ gives rise to so called P-divisible evolution [4], which is characterized by the following conditions

$$\gamma_1(t) + \gamma_2(t) \geq 0, \; \gamma_2(t) + \gamma_3(t) \geq 0, \; \gamma_3(t) + \gamma_1(t) \geq 0, \tag{S8}$$

for all $t \geq 0$. As advocated in Ref. [4], a quantum evolution which violates equation (S5), but satisfies equation (S8), displays only weak non-Markovianity. These examples show that essentially non-Markovian evolution, i.e., the one that violates both equation (S5) and equation (S8), may lead to increase in the extractable work, whereas weakly non-Markovian satisfies $W_{ex}(t_2) \leq W_{ex}(t_1)$ for all $t_1 \leq t_2$.

II. ISING MODEL

As a second example we consider a qubit (S or Q) transversely coupled to an Ising spin chain in a transverse field [5]. Such interaction results in a pure dephasing dynamics of the qubit described by the following master equation [6]

$$L_t(\rho) = i\Lambda(t)[\sigma_z, \rho] + \gamma(t)(\sigma_z\rho\sigma_z - \rho)\,, \tag{S9}$$

with $\Lambda(t)$ being the time-dependent Lamb shift and $\gamma(t)$ a time-dependent decay rate further related to the Loschmidt echo $L(t)$ via relation

$$\gamma(t) = -\frac{\dot{L}(t)}{4L(t)}. \tag{S10}$$

Notice that this corresponds to a special case of a Pauli channel with $\gamma_1(t) = \gamma_2(t) = 0$ and $\gamma_3(t) = \gamma(t)$. Using the equations (S3) and (S7) it is straightforward to show that

$$I(S:Q_t) = 2 + \frac{1}{2}\left(1 - \sqrt{L(t)}\right)\log_2\left(\frac{1}{2}\left(1 - \sqrt{L(t)}\right)\right) + \frac{1}{2}\left(1 + \sqrt{L(t)}\right)\log_2\left(\frac{1}{2}\left(1 + \sqrt{L(t)}\right)\right), \tag{S11}$$

which can be inserted to equation (S6) to obtain the extractable work. For this model, the Loschmidt echo depends on two relevant parameters, λ describing the strength of the transverse field and δ the qubit-environment coupling strength. The analytical expression for the Loschmidt echo is given by

$$L(\lambda, \delta, t) = \Pi_{k>0}\left[1 - \sin^2(2\beta_k)\sin^2(\epsilon_e^k t)\right], \tag{S12}$$

where β_k are the Bogoliubov angles and ϵ_e^k the single quasiparticle excitation energies of the system with the qubit in the state $|1\rangle$; for more details see Ref. [7].

In Fig. 3 of the Article we present three curves describing the time evolution of the extractable work for $\lambda = 1.8$ (orange long-dashed line), $\lambda = 0.0$ (green short-dashed line) and $\lambda = 0.9$ (black solid line). The remaining parameters are fixed for all the plots as $\delta = 0.1$, $J = 1$ and the number of spins $N = 4000$.

III. PHOTONIC BAND GAP

The third physical model that we analyse is the amplitude damping channel described by the following exact local generator:

$$L_t(\rho) = -\frac{is(t)}{2}\left[\sigma_+\sigma_-, \rho\right] + \gamma(t)\Big(\sigma_-\rho\sigma_+ - \frac{1}{2}\{\sigma_+\sigma_-, \rho\}\Big), \tag{S13}$$

with $s(t) = -2\,\mathrm{Im}\left[\dot{G}(t)/G(t)\right]$ and $\gamma(t) = -2\,\mathrm{Re}\left[\dot{G}(t)/G(t)\right]$ the time-dependent Lamb shift and decay rate, respectively. In equation (S13) σ_+ and σ_- stand for raising and lowering operators, respectively, and the complex function $G(t)$ satisfies $|G(t)| < 1$ for all $t > 0$, and directly depends on the features of the reservoir.

The dynamical map of this evolution can be described in terms of the Kraus representation $\Phi_t(\rho) = \sum_{i=1}^2 K_i(t)\rho K_i(t)^\dagger$, where

$$K_1(t) = \begin{pmatrix} 1 & 0 \\ 0 & G(t) \end{pmatrix}, \qquad K_2(t) = \begin{pmatrix} 0 & \sqrt{1 - |G(t)|^2} \\ 0 & 0 \end{pmatrix}. \tag{S14}$$

We consider the model in which the reservoir is an ideal photonic crystal with a frequency band gap [7]. In such a case the specific form of $G(t)$ is as follows:

$$G(t) = 2v_1 x_1 e^{\beta x_1^2 + i\delta t} + v_2(x_2 + y_2)e^{\beta x_2^2 t + i\delta t} - \sum_{j=1}^{3} a_j y_j \left[1 - \Phi(\sqrt{\beta x_j^2 t})\right] e^{\beta x_j^2 t + i\delta t}, \tag{S15}$$

where Φ is an error function. One can see that $G(t)$ depends on two relevant parameters, the detuning $\delta = \omega_0 - \omega_e$ from the band gap edge frequency ω_e, which we set to $\delta = -1$, and

the characteristic frequency parameter β defined as $\beta^{3/2} = \omega_0^{7/2} d^2/6\pi\epsilon_0\hbar c^3$ with ϵ_0 the Coulomb constant and d the atomic dipole moment. In addition:

$$
\begin{aligned}
x_1 &= (A_+ + A_-)e^{i(\pi/4)}, \\
x_2 &= (A_+ e^{-i(\pi/6)} - A_- e^{i(\pi/6)})e^{-i(\pi/4),} \\
x_3 &= (A_+ e^{i(\pi/6)} - A_- e^{-i(\pi/6)})e^{i(3\pi/4)},
\end{aligned}
\tag{S16}
$$

$$
A_\pm = \left[\frac{1}{2} \pm \frac{1}{2}\left[1 + \frac{4}{27}\frac{\delta^3}{\beta^3}\right]^{1/2}\right]^{1/3}, \tag{S17}
$$

$$
v_j = \frac{x_j}{(x_j - x_i)(x_j - x_k)} \quad (j \neq i \neq k; j, i, k = 1, 2, 3), \tag{S18}
$$

$$
y_j = \sqrt{x_j^2} \quad (j = 1, 2, 3). \tag{S19}
$$

Notice that this dynamical map is not unital, so that one does not have the symmetrical situation with respect to the system and the memory dynamics, that was present in the previous models. Hence, it is a good candidate for investigating the counter-intuitive behaviour of work extraction described in the equation (4) of the Article. We therefore focus on the case where the system is subjected to the amplitude damping channel described above. As in previous examples, the initial state is $|SQ\rangle = \frac{1}{\sqrt{2}}(|10\rangle - |01\rangle)$. The entropic quantities entering in the definition of extractable work can be calculated analytically

$$
H(S_t) = -\frac{1}{2}\left(2 - |G(t)|^2\right)\log_2\left(\frac{1}{2}\left((2 - |G(t)|^2\right)\right) - \frac{1}{2}|G(t)|^2 \log_2\left(\frac{1}{2}|G(t)|^2\right), \tag{S20}
$$

$$
H(Q) = 1, \tag{S21}
$$

$$
H(S_tQ) = -\frac{1}{2}\left(1 - |G(t)|^2\right)\log_2\left(\frac{1}{2}\left(1 - |G(t)|^2\right)\right) - \frac{1}{2}\left(1 + |G(t)|^2\right)\log_2\left(\frac{1}{2}\left(1 + |G(t)|^2\right)\right), \tag{S22}
$$

and further used to calculate the mutual information $I(S_t : Q) = H(S_t) + H(Q) - H(S_tQ)$. Using these we get the analytical solution for the extractable work:

$$
W_{ex}(t) = [1 - H(S_t) + I(S_t : Q)]\, kT \ln 2. \tag{S23}
$$

This example, where the system is subjected to the photonic band gap amplitude damping, illustrates that the counterintuitive situation in which one has revivals of extractable work even though the entropy of the system increases, can occur in realistic physical systems; see the shaded

intervals of time in Fig. 4 of the main Article.

---

[1] del Rio, L., Åberg, J., Renner, R., Dahlsten, O. & Vedral, V. The thermodynamic meaning of negative entropy. *Nature* **474,** 61-63 (2011).

[2] Chruściński, D. & Wudarski, F. Non-Markovian random unitary qubit dynamics. *Phys. Lett. A* **377,** 21-22, (2013).

[3] Hall, M. J. W, Cresser, J. D., Li, L. & Andersson, E. Canonical form of master equations and characterization of non-Markovianity. *Phys. Rev. A* **89,** 042120 (2014).

[4] Chruściński, D. & Maniscalco, S. Degree of Non-Markovianity of Quantum Evolution. *Phys. Rev. Lett.* **112**, 120404 (2014).

[5] Quan, H. T., Song, Z., Liu, X. F., Zanardi, P. & Sun, C. P. Decay of Loschmidt Echo Enhanced by Quantum Criticality. *Phys. Rev. Lett.* **96,** 140604 (2006).

[6] Haikka, P., Goold, J., McEndoo, S., Plastina, F. & Maniscalco, S. Non-Markovianity, Loschmidth echo, and criticality: A unified picture. *Phys. Rev. A* **85,** 060101(R) (2012).

[7] John, S. & Quang, T. Spontaneous emission near the edge of a photonic band gap *Phys. Rev. A* **50,** 1764 (1994).
